# Geospatial distribution of intestinal parasitic infections in Rio de Janeiro (Brazil) and its association with social determinants

**DOI:** 10.1371/journal.pntd.0005445

**Published:** 2017-03-08

**Authors:** Clarissa Perez Faria, Graziela Maria Zanini, Gisele Silva Dias, Sidnei da Silva, Marcelo Bessa de Freitas, Ricardo Almendra, Paula Santana, Maria do Céu Sousa

**Affiliations:** 1 Center for Neurosciences and Cell Biology (CNC), University of Coimbra, Coimbra, Portugal; 2 Faculty of Pharmacy, University of Coimbra, Coimbra, Portugal; 3 Evandro Chagas National Institute of Infectious Diseases (INI), Fundação Oswaldo Cruz, Rio de Janeiro, Brazil; 4 National School of Public Health (ENSP), Fundação Oswaldo Cruz, Rio de Janeiro, Brazil; 5 Centre of Studies on Geography and Spatial Planning (CEGOT), Department of Geography and Tourism, University of Coimbra, Coimbra, Portugal; Swiss Tropical and Public Health Institute, SWITZERLAND

## Abstract

**Background:**

Intestinal parasitic infections remain among the most common infectious diseases worldwide. This study aimed to estimate their prevalence and provide a detailed analysis of geographical distribution of intestinal parasites in the metropolitan region of Rio de Janeiro, considering demographic, socio-economic, and epidemiological contextual factors.

**Methods/Principal findings:**

The cross-section survey was conducted among individuals attending the Evandro Chagas National Institute of Infectious Diseases (FIOCRUZ, RJ) during the period from April 2012 to February 2015. Stool samples were collected and processed by sedimentation, flotation, Kato-Katz, Baermann-Moraes and Graham methods, iron haematoxylin staining and safranin staining. Of the 3245 individuals analysed, 569 (17.5%) were infected with at least one parasite. The most common protozoa were *Endolimax nana* (28.8%), *Entamoeba coli* (14.8%), Complex *Entamoeba histolytica/Entamoeba dispar* (13.5%), *Blastocystis hominis* (12.7%), and *Giardia lamblia* (8.1%). *Strongyloides stercoralis* (4.3%), *Schistosoma mansoni* (3.3%), *Ascaris lumbricoides* (1.6%), and hookworms (1.5%) were the most frequent helminths. There was a high frequency of contamination by protozoa (87%), and multiple infections were observed in 141 participants (24.8%). A positive association between age (young children) and gender (male) with intestinal parasites was observed. Geospatial distribution of the detected intestinal parasitic infections was not random or homogeneous, but was influenced by socioeconomic conditions (through the material deprivation index (MDI)). Participants classified in the highest levels of deprivation had higher risk of having intestinal parasites.

**Conclusions/Significance:**

This study provides the first epidemiological information on the prevalence and distribution of intestinal parasitic infections in the Rio de Janeiro metropolitan area. Intestinal parasites, especially protozoa, are highly prevalent, indicating that parasitic infections are still a serious public health problem. MDI showed that intestinal parasites were strongly associated with the socioeconomic status of the population, thus making it possible to identify social vulnerable areas.

## Author summary

Intestinal parasitic infections are considered indicators of health and socio-environmental vulnerability, and are associated with precarious sanitation and water quality of a country. They continue to pose a serious public health problem, especially in developing countries where sanitation is not expanded in line with population growth, such that access to basic services becomes more difficult. Although Brazil is a country with a high prevalence of intestinal parasitic infections, the prevalence in the metropolitan region of Rio de Janeiro (the second largest metropolitan area in the country) has not been estimated. Based on the identification of social determinants (income, education and sanitation), our group was able to identify vulnerable areas for intestinal parasitic infection in the metropolitan region of Rio de Janeiro. Infections caused by intestinal parasites are not included in the list of diseases compulsory notification in Brazil. However, special attention should be focused on this topic, and information on the geographic distribution and prevalence of intestinal parasites, as well as the recognition of vulnerable areas, are the first steps, and a prerequisite for development of appropriate control strategies by the government.

## Introduction

Neglected tropical diseases, including intestinal parasitic infections, are a significant cause of morbidity and mortality in endemic countries [1]. Intestinal parasitic infections have particular relevance as they affect the poorest and most deprived areas in tropical and subtropical regions [1]. It is increasingly recognized that both protozoan and helminthic diseases are common among children under the age of five years. Children are more vulnerable to soil-transmitted helminths (STHs) than adults, and the nutritional impairment caused by the parasite can lead to iron-deficiency anaemia, malnutrition, and a negative impact on growth and cognitive development [2,3].

Despite all the medical and pharmaceutical advances and developments in sanitary engineering, intestinal parasitic infections remain among the most common infectious diseases worldwide, particularly in developing countries, where inadequate water treatment, poor sanitation and lack of adequate health services are common. Additionally, it is more difficult to implement enteric parasite-control actions in these regions due to the high cost of improvements in infrastructure, and the lack of educational projects offered to the population [1,4,5].

Water is essential to life, but is also a major vehicle for pathogen dissemination. The potential for waterborne parasite transmission is high since infective helminth eggs and protozoa (oo)cysts are distributed through water in the environment. Pathogens like *Giardia lambia* and *Cryptosporidium* spp. are recognized as important waterborne disease pathogens and are associated with severe gastrointestinal illness. Amoebiasis, balantidiosis, cyclosporidiosis and microsporidiosis outbreaks have been reported throughout the world [6,7]. It is well documented that conventional water and sewage treatment process are not completely effective in destroying protozoa (oo)cysts and helminth eggs [8–10]. Improper disposal of human and animal waste has also been identified as a source of infection, contaminating water sources [11] and recreational waters such as swimming pools, water parks and lakes [9]. Occasionally, sewer overflows also contribute to contamination of surface water and agricultural lands, which leads to potential human infection. Food contamination is also important and can occur directly in the handling process (contaminated equipment, infected food handlers or wash water), or indirectly through contaminated irrigation water [12].

The lack of sanitary conditions to which the population is exposed favours the acquisition of various pathogens, and patients are often multiply infected (polyparasitized). Recently, a systematic review and meta-analysis showed that sanitation facilities and water treatment are associated with lower risks of infection with intestinal protozoa, and could also prevent diarrhoeal diseases [1]. The same relationships were observed by Strunz *et al*. [13] for soil-transmitted helminths.

In Brazil, intestinal parasite infections persist, although their frequency has decreased due improvement of sanitary conditions [14–16]. Up until now, studies of enteric parasites in Brazil have been limited, isolated and fairly rare, generally reflecting the situation in small towns. Mariano and colleagues [17] observed 77.2% of positive cases, and a polyparasitism of 51.2% in children from Itabuna (Bahia). Similar results were observed in two localities of São Paulo, where 65.9% of the individuals were positive for at least one parasite [18]. In Rio de Janeiro, previous studies have shown intestinal parasite prevalence ranging from 18.3% to 66% [19–24].

The aim of this study was to estimate the number of individuals infected with intestinal parasites who attended a referral hospital located in Rio de Janeiro (Brazil), and to provide a detailed analysis of the geographical distribution. The study also looked at the influence of demographic variables, socio-economic status and environmental factors on the intestinal parasitic infections. This knowledge will be essential for the development of effective prevention and control strategies to eliminate or reduce intestinal parasitic infection.

## Methods

### Ethics statement

The Research Ethics Committee Evandro Chagas National Institute of Infectious Diseases (INI/FIOCRUZ) approved the study (protocol number: 127.542). This project was in accordance with the Brazilian Ethical Resolutions, especially Resolution CNS 196/1996 and its complementary and the Code of Medical Ethics of 1988 (articles 122–1307). Study individuals provided a written signed informed consent prior to sample collection and for participants younger than 18 years, informed consent was provided by parents or guardians after a detailed explanation of the objectives of the work. A term of privacy and confidentiality was signed by the researches for individuals to whom it was not possible to obtain informed consent beforehand.

### Study site

The cross-section survey was carried out from April 2012 to February 2015 in Evandro Chagas National Institute of Infectious Diseases (INI/FIOCRUZ), a reference hospital in infectious diseases in Brazil, located in Rio de Janeiro (RJ). Despite it being an infectious disease referral hospital, individuals also attend for routine consultations (cardiology, dermatologist, gynecology, neurology, ophthalmology, otolaryngologist, infectious disease speciality) or emergency situations. As the prevalence of intestinal parasites in Brazil remains high, it is common the doctor´s submit requests for parasitological analysis in faeces, regardless of age or genera and of having or not symptoms suggestive of intestinal infections. The INI/FIOCRUZ hospital receives individuals from all municipalities, mainly the metropolitan area.

**Fig 1:**
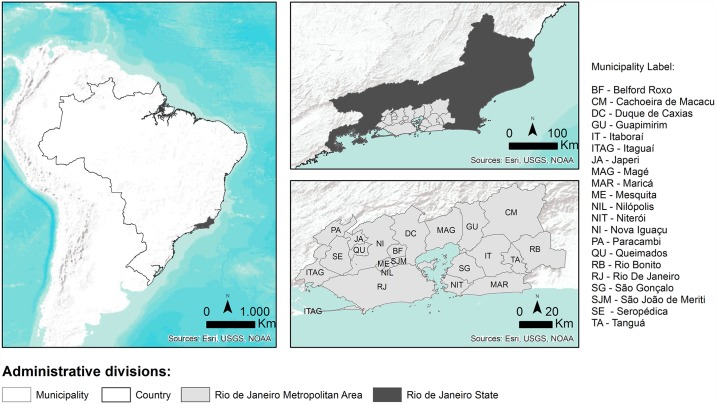
Localization of the metropolitan region of Rio de Janeiro state, Brazil.

Rio de Janeiro State is composed of 92 municipalities. The metropolitan region of Rio de Janeiro is composed of 21 municipalities: Belford Roxo, Cachoeira de Macacu, Duque de Caxias, Guapimirim, Itaboraí, Itaguaí, Japeri, Magé, Maricá, Mesquita, Nilópolis, Niterói, Nova Iguaçu, Paracambi, Queimados, Rio Bonito, Rio de Janeiro, São Gonçalo, São João de Meriti, Seropédica and Tanguá (Fig 1). It is the second largest metropolitan area in Brazil with 11.812.482 inhabitants in an area of 8.147.356 km^2^. This region has 2.746 slums, with a resident population of 1.702.073 inhabitants (14.4% from the total population) occupying 123.627km^2^ [25]. The main characteristics of each municipality of the metropolitan region of Rio de Janeiro State are summarized in Table 1.

**Table 1:** Main characteristics of municipalities of the metropolitan region of Rio de Janeiro state.

Municipalities	Population*	Population density (inhab./km^2^)*	Drinking water coverage (%)*	Sanitation coverage (%)*	MHDI**	Gini coefficient*
Belford Roxo	469.332	6.031.38	76.8	39.3	0.684	0.4606
Cachoeira de Macacu	54.273	56.90	94.8	86.5	0.7	0.5077
Duque de Caxias	855.048	1.828.51	85.1	41.6	0.711	0.4875
Guapimirim	51.483	1.142.70	43.9	-	0.698	0.5232
Itaboraí	218.008	506.56	81.7	40.3	0.693	0.4967
Itaguaí	109.091	395.45	86.4	37.0	0.715	0.5004
Japeri	95.492	1.166.37	67.2	-	0.659	0.4578
Magé	227.322	585.13	79.7	40.6	0.709	0.5082
Maricá	127.461	351.55	58	12.3	0.765	0.5098
Mesquita	168.376	4.310.48	82.6	37.2	0.737	0.4919
Nilópolis	157.425	8.117.62	98.3	95.9	0.753	0.4805
Niterói	487.562	3.640.80	100	92.7	0.837	0.5983
Nova Iguaçu	796.257	1.527.60	92.1	42.0	0.713	0.5141
Paracambi	47.124	262.27	73.1	29.9	0.72	0.4718
Queimados	137.962	1.822.60	79.7	37.0	0.68	0.4584
Rio Bonito	55.551	121.70	87.2	-	0.71	0.5023
Rio de Janeiro	6.320.446	5.265.81	91.2	70.1	0.799	0.6391
São Gonçalo	999.728	4.035.90	85.1	36.8	0.739	0.4610
São João de Meriti	458.673	13.024.56	91.8	48.7	0.719	0.4620
Seropédica	78.186	275.53	69.6	31.1	0.713	0.4835
Tanguá	30.732	211.21	68.3	29.9	0.654	0.4615

* [25]

** [26]

According to the last census conducted in 2010, Rio de Janeiro municipality has a population of 6.320.446 inhabitants (Table 1) in an area of 1.197.463 km^2^. The municipality has 2.227 slums, with a resident population of 1.393.314 inhabitants (11.8% from the total population) occupying 54.213 km^2^ [25].

Municipal human development index (MHDI) is a summary measure of average achievement in key dimensions of human development (a long and healthy life, being knowledgeable and have a decent standard of living), and gini index is a measure of statistical dispersion whose value ranges from zero (perfect equality) to one (perfect inequality). The MHDI of Rio de Janeiro is 0.799 according to the United Nations Development Programme [26] and gini index is 0.6391 [25]. Most of the population (91.2%) has access to potable water and 70.1% has sanitation coverage [27].

### Study population, collection of faecal samples and laboratory methods

The study population included individuals (n = 3245), of both genders and all age groups, attended in Evandro Chagas National Institute of Infectious Diseases, between April 2012 and February 2015. Stool samples were collected by the participant in plastic disposable flasks with or without preservatives and maintained at 4°C until laboratory analysis on the same day. Flasks were labelled with the name, collection date and the hospital number. The parasitological tests were conducted at the Parasitology Laboratory of INI by experienced laboratory technologists and College of American Pathologist certifies the Laboratory. Moreover, participant’ data (sex, age, educational level and residence) were obtained from the hospital’s database.

For laboratory diagnosis of intestinal parasites, the fresh specimens were analysed by means of centrifugation sedimentation [28], centrifugal flotation in zinc sulphate solution [29], Kato-Katz (Helm-TEST kit, Fiocruz, Brazil) and Baermann-Moraes method [28,30]. All these techniques were routinely performed on all fresh stool samples. Specimens preserved in MIF solution (mertiolate-iodine-formaldehyde) were processed by the centrifugation sedimentation method [28]. The Graham method, faecal occult blood test, the iron haematoxylin staining and the safranin staining procedure was carried out depending on doctor request [28]. The slides were then observed under the optical microscope.

All individuals attended in INI/FIOCRUZ are dewormed when diagnosed (drugs are provided by the institution itself).

### Data extraction and geospatial analysis

The zip code for each participant was obtained from the hospital’s database and through Brazilian Institute of Statistics and Geography (IBGE) converted into geographic coordinates (latitude and longitude). IBGE was the source of data in respect of geography, demography and socioeconomic conditions of the studied population (National Census of 2010).

The spatial distribution of the participants was assessed through a Kernel Density Function that allows to estimate the intensity of events across a surface by calculating the overall number of cases within a given search radius from a target point. To identify if the participants were spatially clustered or dispersed the Average Nearest Neighbor test was used.

To evaluate the social and economic conditions of the place of residence a material deprivation index (MDI) was constructed, at the census tract level, to the metropolitan region of Rio de Janeiro. The MDI is based upon the following indicators: (1) illiteracy rate/education (percentage of population older than 10 years that can read or write); (2) water supply/sanitation (percentage of permanent households without public water treatment plant); and (3) family income (percentage of households with per capita monthly income ≤1 minimum wage). Based on the Carstairs and Morris method, the indicators considered in each index were standardised (using the z-score method) so that each indicator has a weighted mean of zero and a variance of one, and exerted the same influence upon the final result [31]. The MDI was analysed in quintiles: q1, lowest level of deprivation; q5, highest level of deprivation.

To address the potential effects of the socioeconomic conditions of the place of residence on the incidence of intestinal parasites, the proportion of participants living in each deprivation quintile was assessed. Simultaneously, the proximity to slums was analysed through geographical buffers of 50m and 100m. The spatial analysis was performed through the ArcMap 10.x software of ESRI.

### Statistical analysis

The data entry was carried out using Excel software and analysed using Statistical Package for the Social Sciences (SPSS) version 16. Percentages were used to perform the exploratory analysis of the categorical variables and quantitative variables are presented as mean ± standard deviation (SD). Pearson´s chi-squared and Fisher’s Exact Test were used for categorical data. The level of statistical significance was set as p<0.05, an odds ratio and 95% confidence interval (CI) was computed. Logistic regression was used to identify a potential contribution of each of the variables for the acquisition of intestinal parasite infections.

## Results

### Prevalence of intestinal parasitic infections

Between April 2012 and February 2015, a total of 3245 individuals (1564 female and 1681 male) had the parasitological tests done (Table 2). In 2012 a total of 995 samples were collected, with 193 positive samples; in 2013, 1189 individuals were collected being 187 positive samples; in 2014, 938 individuals with 168 positive samples; and in 2015, 123 individuals with 21 positive samples. Summarizing, we had 569 individuals (17.5%) with positive stool examination for one or more enteric parasite and 2676 individuals (82.5%) with negative results.

**Table 2:** Prevalence of intestinal parasites along the years of the survey.

Year of collection	Positive participants No. (%)	Negative participants No. (%)	Total
2012	193 (19.4)	802 (80.6)	995 (30.7)
2013	187 (15.7)	1002 (84.3)	1189 (36.6)
2014	168 (17.9)	770 (82.1)	938 (28.9)
2015	21 (17.1)	102 (82.9)	123 (3.8)
**Total**	**569 (17.5)**	**2676 (82.5)**	**3245 (100)**

The ages of the participants ranged from 1 to 93 years with an average of 41.34±15.54 (Mean±SD; median = 41). The adults between 26–65 years were the majority of participants (n = 2130) (Table 3). There were more male than female parasitized (64.5% versus 35.5%, respectively) and seventy-five percent of participants (n = 427) were educated above the primary grade (Table 3).

**Table 3:** Characteristics of positive and negative participants to intestinal parasites.

Characteristics	No. (%) positive participants (n = 569)	No. (%) negative participants (n = 2676)	Total (n = 3245)
**Age group (years)**			
0–14	16 (23.9)	51 (76.1)	67 (2.1)
15–25	81 (18.3)	361 (81.7)	442 (13.6)
26–65	417 (19.6)	1714 (80.4)	2131 (65.7)
>66	25 (13.6)	159 (80.5)	184 (5.7)
Missing	30 (7.1)	391 (92.9)	421 (12.9)
**Gender**			
Female	202 (12.9)	1362 (87.1)	1564 (48.2)
Male	367 (21.8)	1314 (78.2)	1681 (51.8)
**Education**			
Elementary school	190 (33.4)	na	na
High school	152 (26.7)	na	na
University education	85 (14.9)	na	na
No formal education	17 (3)	na	na
Missing	125 (22)	na	na

* *na*, not available

*Endolimax nana* was the most common enteric parasite, present in 216 samples (28.8%) followed by *Entamoeba coli* in 111 samples (14.8%), Complex *Entamoeba histolytica/Entamoeba dispar* in 101 samples (13.5%), *Blastocystis hominis* in 95 samples (12.7%), *Giardia lamblia* in 61 samples (8.1%), *Iodamoeba butschilii* in 33 samples (4.4%), *Strongyloides stercoralis* in 32 samples (4.3%), *Schistosoma mansoni* in 25 samples (3.3%), *Cryptosporidium sp*. in 14 samples (1.9%), *Ascaris lumbricoides* in 12 samples (1.6%), *Cystoisospora belli* in 12 samples (1.6%), *hookworms* in 11 samples (1.5%), *Trichuris trichiura* in 10 samples (1.3%), *Entamoeba hartmani* in 9 samples (1.2%), *Enterobius vermicularis* in 6 samples (0.8%) and *Hymenolepis nana* in one sample (0.1%) (Table 4).

**Table 4:** Number of intestinal protozoa and helminths species: Monoparasitism and polyparasitism.

Parasites	No (%) (n = 749)	Monoparasitism (n = 428)	Polyparasitism (n = 321)
**Protozoa**			
*B*. hominis***	95 (12.7)	66 (15.4)	29 (9)
Complex *E*. *histolytica/ E*. dispar*	101 (13.5)	50 (11.7)	51 (15.9)
*Cryptosporidium sp*.*	14 (1.9)	11 (2.6)	3 (0.9)
*C*. belli*	12 (1.6)	11 (2.6)	1 (0.3)
*E*. nana**	216 (28.8)	141 (32.9)	75 (23.4)
*E*. coli**	111 (14.8)	41 (9.6)	70 (21.8)
*E*. hartmani**	9 (1.2)	3 (0.7)	6 (1.9)
*G*. lamblia*	61 (8.1)	36 (8.4)	25 (7.8)
*I*. butschilii**	33 (4.4)	7 (1.6)	26 (8.1)
Total of protozoa species	652 (87)	366 (85.5)	286 (89)
**Helminths**			
*A*. lumbricoides*	12 (1.6)	4 (0.9)	8 (2.5)
*E*. vermicularis*	6 (0.8)	5 (1.2)	1 (0.3)
*H*. nana*	1 (0.1)	-	1 (0.3)
Hookworms*	11 (1.5)	6 (1.4)	5 (1.6)
*S*. mansoni*	25 (3.3)	20 (4.7)	5 (1.6)
*S*. stercoralis*	32 (4.3)	23 (5.4)	9 (2.8)
*T*. trichiura*	10 (1.3)	4 (0.9)	6 (1.9)
Total of helminths species	97 (13)	62 (14.5)	35 (11)

* Pathogenic species;

** Non-pathogenic species;

*** Non-pathogenic, human pathogen that remain unclear.

The number of samples with one parasite (monoparasitism) is higher (428 positive samples, 57.1%) than those samples with various parasites (polyparasitism) (321 positive samples, 42.9%). Interesting, the frequency of the amoebae (Complex *E*.*histolytica*/*E*.*dispar*, *E*. *coli* and *E*. *hartmani)* as well of some geohelminths (*A*. *lumbricoides* and *T*. *trichiura*) is higher on samples with various parasites (polyparasitism) (Table 4).

We observed a very high frequency of protozoan infections (87%), occupying the first six positions; *E*. *nana* was the predominant, followed by *E*. *coli* and Complex *E*. *histolytica/E*. *dispar*. The most frequent helminths were *S*. *stercoralis* and *S*. *mansoni;* only appearing in seventh position. Of the 16 species of intestinal parasites detected, 11 were pathogenic (Complex *E*. *histolytica/E*. *dispar*, *Cryptosporidium sp*., *C*. *belli*, *G*. *lamblia*, *A*. *lumbricoides*, *E*. *vermicularis*, *H*. *nana*, *hookworms*, *S*. *mansoni*, *S*. *stercoralis* and *T*. *trichiura*) and 5 were non-pathogenic (*B*. *hominis*, *E*. *nana*, *E*. *coli*, *E*. *hartmani* and *I*. *butschilii*). The pathogenic species comprises 38.1% of the studied participants (285 of 749), while the non-pathogenic reached 61.9% (464 of 749).

Most of the participants (428 of 569; 75.2%) did not present any co-infection, whereas 141 (24.8%) had two or more parasites simultaneously. Among the multiple infected, 109 individuals were infected with two parasites (19.2%), 26 were infected with three parasites (4.6%), 5 had four parasites (0.9%) and 1 had five (0.1%). Regarding parasitic associations, only 11.8% (67 of 569) were co-parasited by helminths, 84.3% (480 of 569) by protozoa and only 3.9% (22 of 569) by both.

### Intestinal parasites risk factors

Age and gender were examined as potential associations for intestinal parasitic infections. A positive association between gender and intestinal parasites (*p*<0.0001), as well as protozoa (*p*<0.0001), helminths (*p*<0.0001) and poliparasitism (*p*<0.0001) were detected. Male were more likely to be infected with intestinal parasites (OR = 1.9; 95%CI of 1.56 to 2.27), protozoa (OR = 1.8; 95%CI of 1.50 to 2.20), helminths (OR = 2.8; 95%CI of 1.75 to 4.51) and have multiple parasites (OR = 3.4; 95% CI of 2.28 to 5.05) compared to female (Table 5).

**Table 5:** Associations of intestinal parasites with the gender.

Parasites	Gender		OR (95%CI)	*p*-value
	Male (n = 1681)	Female (n = 1564)		
	No. (%)	No. (%)		
Intestinal parasites	367 (21.8)	202 (12.9)	1.9 (1.56; 2.27)	0.0001
Protozoa	302 (17.9)	182 (11.6)	1.8 (1.50; 2.20)	0.0001
Helminths	65 (3.9)	24 (1.5)	2.8 (1.75; 4.51)	0.0001
Monoparasitism	259 (15.4)	169 (10.8)	1.6 (1.29; 1.96)	0.0001
Polyparasitism	108 (6.4)	33 (2.1)	3.4 (2.28; 5.05)	0.0001

No statistical significant difference was found between intestinal parasites and age (*p* = 0.166). However, when we analyse the parasite species separately we observed that children (0–14 years) were more likely to be infected with *A*. *lumbricoides* (*p* = 0.031; OR = 8.5; 95% CI = 1.8; 39.4), *E*. *vermicularis* (*p* = 0.005; OR = 28.2; 95% CI = 4.6; 171.6), *B*. *hominis* (*p* = 0.002; OR = 3.9; 95% CI = 1.8; 8.4), and *G*. *lamblia* (*p* = 0.011; OR = 4.1; 95% CI = 1.6; 10.7) as compared to the older participants (S1 Table). Moreover, there were no cases of multiple parasitic infections in children under 5 years old (S1 Table).

### Geospatial distribution

The prevalence of intestinal parasites varies by municipalities, most of participants (2847 of 3245; 87.7%) live in metropolitan region and 1748 (53.9%) live in Rio de Janeiro municipality (Tables 6 and S2). The metropolitan region of Rio de Janeiro had 532 positive cases (16.4%) and the others municipalities had 21 positive cases (0.6%) (Table 6). As expected, Rio de Janeiro municipality had a greater number of participants infected with intestinal parasites (332; 10.2%) since it has the larger population (S2 Table, S1 Fig). In 16 participants (0.5%) positive for intestinal parasites was not possible to identify the residence.

**Table 6:** Number of positive and negative participants to intestinal parasites by regions.

Regions	Positive participants No. (%)	Negative participants No. (%)	Total of participants No. (%)	Prevalence rates
**Rio de Janeiro State**				
Metropolitan region	532 (16.4)	2315 (71.3)	2847 (87.7)	18.7
Others municipalities	21 (0.7)	60 (1.8)	81 (2.5)	26.6
**Others States of Brazil**	-	9 (0.3)	9 (0.3)	-
Missing	16 (0.5)	292 (9)	308 (9.5)	5.2
**Total**	**569**	**2676**	**3245**	**17.5**

The distribution of parasites species also varied among the municipalities (Table 7). The metropolitan region had 93.7% (702 of 749) of the enteric parasites observed: in Rio de Janeiro it was possible to detect 434 enteric parasites (57.9%), Duque de Caxias was the second municipality with 81 (10.8%), followed by Nova Iguaçu (57; 7.6%), Belford Roxo (33; 4.5%), São João de Meriti (25; 3.4%), São Gonçalo (18; 2.4%), Nilópolis (15; 2%), Magé (12; 1.6%), Cachoeira de Macacu (5; 0.7%), Itaboraí (4; 0.5%), Niterói (3; 0.4%), Queimados (3; 0.4%), Itaguaí (2; 0.3%), Maricá (2; 0.3%), Mesquita (2; 0.3%), Seropédica (2; 0.3%), and Japeri (4; 0.5%). We did not have positive samples from participants of Guapimirim, Paracambi, Rio Bonito and Tanguá. Others municipalities amounted 28 (3.7%) enteric parasites, and 19 (2.5%) was not possible to identify the municipality (Table 7).

**Table 7:** Distribution of intestinal parasites species by the municipalities of Rio de Janeiro state.

Municipality	Number (%) of intestinal parasites species																Total
	Bh	Eh/Ed	Crypto	Cb	En	Ec	Eh	Gl	Ib	Al	Ev	Hk	Hn	Sm	Ss	Tt	
**Metropolitan Region**	93 (12.4)	93 (12.4)	14 (1.9)	12 (1.6)	198 (26.4)	104 (13.9)	7 (1.0)	57 (7.6)	32 (4.3)	12 (1.6)	6 (0.8)	10 (1.4)	1 (0.1)	25 (3.4)	30 (4.0)	8 (1.1)	702 (93.7)
Belford Roxo	4 (0.5)	4 (0.5)			8 (1.1)	7 (1.0)	2 (0.3)	2 (0.3)	3 (0.4)			1 (0.1)		2 (0.3)			33 (4.5)
Cachoeira de Macacu	1 (0.1)				2 (0.3)	2 (0.3)											5 (0.7)
Duque de Caxias	13 (1.8)	9 (1.2)	1 (0.1)		19 (2.6)	11 (1.5)	1 (0.1)	12 (1.6)	4 (0.5)	2 (0.3)		1 (0.1)		2 (0.3)	3 (0.4)	3 (0.4)	81 (10.8)
Itaboraí	1 (0.1)				1 (0.1)	1 (0.1)				1 (0.1)							4 (0.5)
Itaguaí	1 (0.1)														1 (0.1)		2 (0.3)
Japeri	1 (0.1)	1 (0.1)			1 (0.1)				1 (0.1)								4 (0.5)
Magé	2 (0.3)	2 (0.3)		1 (0.1)	3 (0.4)	1 (0.1)				1 (0.1)	1 (0.1)					1 (0.1)	12 (1.6)
Maricá					1 (0.1)	1 (0.1)											2 (0.3)
Mesquita				1 (0.1)	1 (0.1)												2 (0.3)
Nilópolis	3 (0.4)	2 (0.3)	1 (0.1)		2 (0.3)	1 (0.1)		2 (0.3)	1 (0.1)	1 (0.1)				1 (0.1)	1 (0.1)		15 (2.0)
Niterói			1 (0.1)		2 (0.3)												3 (0.4)
Nova Iguaçu	6 (0.8)	9 (1.2)	2 (0.3)		14 (1.9)	8 (1.1)	1 (0.1)	6 (0.8)	2 (0.3)	1 (0.1)		1 (0.1)		2 (0.3)	4 (0.5)	1 (0.1)	57 (7.6)
Queimados		1 (0.1)				1 (0.1)								1 (0.1)			3 (0.4)
Rio de Janeiro	58 (7.7)	59 (8.1)	7 (1.0)	10 (1.4)	127 (17.4)	63 (8.6)	3 (0.4)	33 (4.4)	18 (2.5)	5 (0.7)	5 (0.7)	7 (1.0)	1 (0.1)	17 (2.3)	18 (2.5)	3(0.4)	434 (57.9)
São Gonçalo	2 (0.3)	3 (0.4)	1 (0.1)		6 (0.8)	3 (0.4)		1 (0.1)	2 (0.3)								18 (2.4)
São João de Meriti	1 (0.1)	3 (0.4)			11 (1.5)	4 (0.5)		1 (0.1)	1 (0.1)	1 (0.1)					3 (0.4)		25 (3.4)
Seropédica			1 (0.1)			1 (0.1)											2 (0.3)
Guapimirim, Paracambi, Rio Bonito and Tanguá	-	-	-		-	-		-	-	-	-	-	-	-	-	-	-
**Others municipalities**	2 (0.3)	4 (0.5)			11 (1.5)	4 (0.5)	2 (0.3)	2 (0.3)							2 (0.3)	1 (0.1)	28 (3.8)
Angra dos Reis							1 (0.1)										1 (0.1)
Araruama		1 (0.1)			3 (0.4)	2 (0.3)											6 (0.8)
Barra Mansa		1 (0.1)													1 (0.1)		2 (0.3)
Comendador Levy Gasparian															1 (0.1)		1 (0.1)
Macaé					1 (0.1)												1 (0.1)
Mangaratiba					2 (0.2)												2 (0.3)
Parati	1 (0.1)																1 (0.1)
Santo Antônio de Pádua					1 (0.1)			2 (0.3)									3 (0.4)
São Pedro da Aldeia						1 (0.1)	1 (0.1)										2 (0.3)
Saquarema	1 (0.1)	1 (0.1)			2 (0.3)	1 (0.1)										1 (0.1)	6 (0.8)
Três Rios		1 (0.1)															1 (0.1)
Valença					1 (0.1)												1 (0.1)
Volta Redonda					1 (0.1)												1 (0.1)
**Unknown**		4 (0.5)			7 (1.0)	3 (0.4)		2 (0.3)	1 (0.1)			1 (0.1)				1 (0.1)	19 (2.5)
**Total**	**95 (12.7)**	**101 (13.5)**	**14 (1.9)**	**12 (1.6)**	**216 (28.8)**	**111 (14.8)**	**9 (1.2)**	**61 (8.1)**	**33 (4.4)**	**12 (1.6)**	**6 (0.8)**	**11 (1.5)**	**1 (0.1)**	**25 (3.3)**	**32 (4.3)**	**10 (1.3)**	**749 (100)**

*Ascaris lumbricoides* (Al), *Enterobius vermicularis* (Ev), Hookworms (Hk), *Hymenolepis nana* (Hn), *Schistosoma mansoni* (Sm), *Strongyloides stercoralis* (Ss), *Trichuris trichiura* (Tt), *Blastocystis hominis* (Bh), Complex E*ntamoeba histolytica/Entamoeba díspar* (Eh/Ed), *Cryptosporidium sp*.(Crypto), *Cystoisospora belli* (Cb), *Endolimax nana* (En), *Entamoeba coli* (Ec), *Entamoeba hartmani* (Eh), *Giardia lamblia* (Gl), *Iodamoeba butschilii* (Ib).

#### Metropolitan region of Rio de Janeiro state

**Fig 2:**
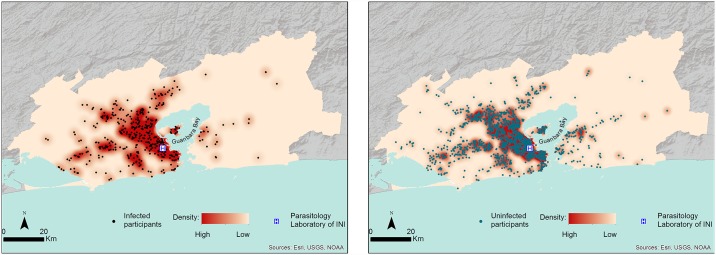
Geographical distribution and gaussian kernel density surface map of participants with and without intestinal parasites. Incidence density in the metropolitan region of Rio de Janeiro State.

Of the 3245 participants, only 2670 informed the zip code that was converted into geographic coordinates. Two thousand six hundred fifty-two (472 infected participants and 2180 participants with negative results) lived in the metropolitan area of Rio de Janeiro State and 1638 (286 infected participants and 1352 participants with negative results) in Rio de Janeiro municipality (S1 Table). The geospatial distribution of participants infected and uninfected by intestinal parasites in the metropolitan region could be observed in Fig 2. Based on participants’ place of residence we could observe a marked geographical pattern, with a high incidence density near Evandro Chagas National Institute of Infectious Diseases and along Guanabara Bay (Fig 2). The geographical distribution of participants with and without intestinal parasites was similar, and we could observe a statistically significant spatial dependency.

**Fig 3:**
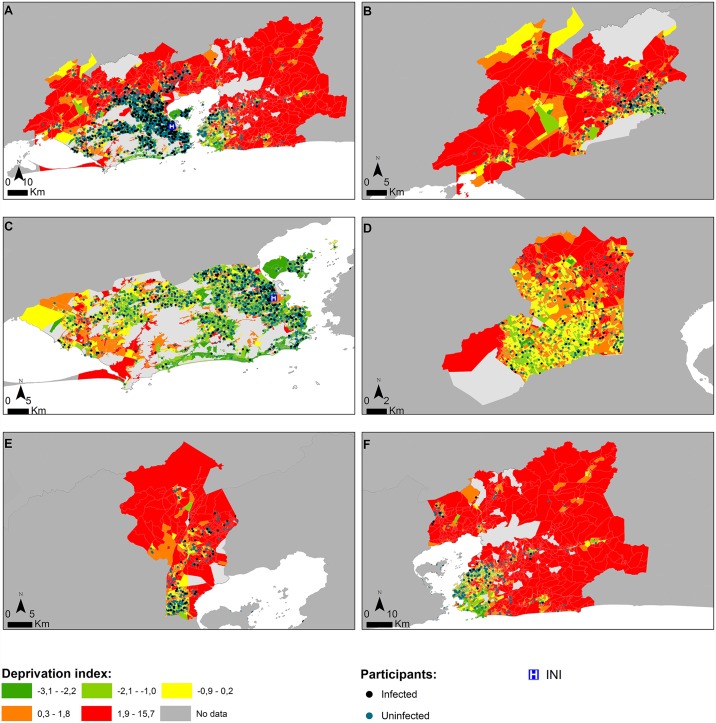
Geographical distribution of participants with and without intestinal parasitic infections and the Material Deprivation Index (MDIs). (A) Metropolitan region of Rio de Janeiro; (B) Itaguaí, Japeri, Queimados, Nova Iguaçu, Paracambi and Seropédica municipalities; (C) Rio de Janeiro municipality; (D) Belford Roxo, Mesquita, Nilópolis and São João de Meriti municipalities; (E) Duque de Caxias municipality; (F) Cachoeira de Macacu, Guapimirim, Itaboraí, Magé, Maricá, Niterói, Rio Bonito, São Gonçalo and Tanguá municipalities (green (q1): lower material deprivation; red (q5): higher material deprivation).

Socioeconomic deprived regions were identified in the metropolitan region of Rio de Janeiro State. Lower scores in the deprivation index (q1) represented lower socioeconomic deprivation, and higher scores in the deprivation index (q5) represented higher socioeconomic deprivation (Fig 3).

Demographic and socioeconomic characteristics considerably varied among the census tracts (CTs) in metropolitan region of Rio de Janeiro (Fig 3). It was possible to observe contiguous CTs with very different material deprivation index (MDIs) (q1 and q5, for example). The number of infected participants was lower in areas with better socioeconomic, educational and sanitary conditions (q1); and a higher number of infected participants were observed in the highest levels of deprivation (q3, q4 and q5). A gradient could be observed between the quintiles (Table 8). Thus, individuals classified in the first deprivation quintile (q1) had less risk of having intestinal parasites than individuals classified in the others deprivation quintiles (Table 8).

**Table 8:** Number of positive and negative participants to intestinal parasites per quintile of deprivation.

Quintiles	Material Deprivation Index (%)			OR (95%CI)	*p*-value
	Positive participants (n = 485)	Negative participants (n = 2185)	Total of participants (n = 2670)		
**Q1**	40 (6.9)	538 (93.1)	578	^§^	0.001*
**Q2**	95 (19.7)	388 (80.3)	483	3.3 (2.2; 4.9)	0.001*
**Q3**	102 (21.4)	374 (78.6)	476	3.7 (2.5; 5.5)	0.001*
**Q4**	117 (25.6)	340 (74.4)	457	4.6 (3.2; 6.9)	0.001*
**Q5**	129 (19.7)	526 (80.3)	655	3.3 (2.3; 4.8)	0.001*
**ND**^†^	2 (9.5)	19 (90.5)	21	-	-

^§^ Reference group

* Statistically significant (p<0.05)

^†^ CTs that were not possible to calculate the MDI

#### Rio de Janeiro municipality

**Fig 4:**
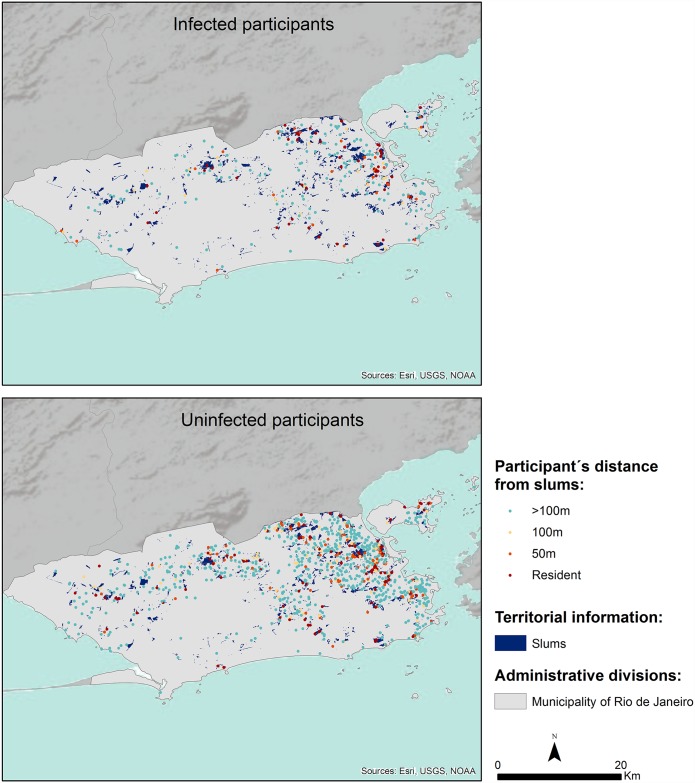
Geographical distribution of participants with and without intestinal parasitic infections and their distance over the slums in Rio de Janeiro municipality.

In Table 9 and Fig 4 we could observe that 65.8% (1078 of 1638) of participants live more than 100 meters away from slums and that 19.2% (314 of 1638) of participants live within a radius of 100 meters to slums. We could also note that 21.3% (61 of 286) of individuals positive for intestinal parasites live in slums (also called, subnormal agglomerates), with inadequate infrastructure and lack of access to health services. Therefore, participants who live more than 100 meters away from slums had less risk of having intestinal parasites (*p* = 0.001) than participants living in slums (*p* = 0.001).

**Table 9:** Number of positive and negative participants to intestinal parasites according to their distance over the slums in Rio de Janeiro municipality.

Participant´s distance from slums (meters)	Positive participants (n = 286)	Negative participants (n = 1352)	Total of participants (n = 1638)	OR (95%CI)	*p*-value
>100	166 (15.4)	912 (84.6)	1078	^§^	0.001*
50–100	25 (16.7)	125 (83.3)	150	1.1 (0.7; 1.7)	0.084
<50	34 (20.7)	130 (79.3)	164	1.4 (0.9; 2.1)	0.688
Resident	61 (24.8)	185 (75.2)	246	1.8 (1.3; 2.5)	0.001*

^§^ Reference group

* Statistically significant (p<0.05)

## Discussion

The current study estimated the prevalence of intestinal parasitic infections among individuals from Rio de Janeiro State (Brazil), in addition to evaluating some epidemiological aspects. Spatial analysis was applied for the first time to the case of Rio de Janeiro to describe the geographical distribution of individuals with enteric parasites infections. The study also looked at socio-economic indicators (social vulnerability indicator) for intestinal infections, in particular family income, education and sanitation (access to safe drinking water). The construction of a material deprivation index allowed us to identify the most vulnerable regions for intestinal parasitic infections in the metropolitan area of Rio de Janeiro State.

The mean prevalence of intestinal parasitic infections remains high in Rio de Janeiro State (17.5%) and also in the metropolitan region and the municipality (18.7% and 19%, respectively). Previous studies suggest that we may observe a decrease in the prevalence of intestinal parasites in Rio de Janeiro with time. A parasitological survey carried out in 1984 on children from day-care centres detected a prevalence of 35% [19]. Further studies carried out on pregnant women [20], children living in low income communities [21] and day-care centres located in slums in the municipality [22] showed a prevalence ranging from 37.6% to 54.5%. A survey made in 2007 in a paediatric hospital [23] detected values of 18.3%. Although our results indicate that the mean prevalence is similar to this last study, it should be noted that individuals attending the Evandro Chagas National Institute of Infectious Diseases (INI/FIOCRUZ) were mainly adults, where it was expected that prevalence would be lower when compared to studies on children.

Age is an important risk factor for intestinal parasitic infections. Children are more susceptible to intestinal infectious diseases than adults because of their poor hygiene habits; they are often in contact with contaminated soil and their immune system is immature [2,32]. In spite of our small number of samples from young participants, we observed a positive association between infections with *A*. *lumbricoides*, *E*. *vermicularis*, *B*. *hominis* and *G*. *lamblia* and the younger age.

The distribution of intestinal parasites varied among the municipalities that compose Rio de Janeiro State, with the highest incidence density of intestinal parasites in municipalities with larger population (Rio de Janeiro, Duque de Caxias, Nova Iguaçu, Belford Roxo and São João de Meriti). These results could be explained by the ease of access to the INI hospital, since these areas have the main road corridors of the municipality (Brazil Avenue, Governador Carlos Lacerda Avenue, Presidente João Goulart Avenue and Presidente Dutra highway), and also because many of the infected population lives near INI hospital. Despite São Gonçalo is the second largest municipality, only 2.4% (18 of 749) of the intestinal parasites were detected there. This municipality is located across the Guanabara Bay, such that access by participants to the INI hospital is probably limited by poor public transportation.

The prevalence of enteric parasites varies between regions of Brazil, and contrasting data are observed: 11.3% in Sergipe [33]; 42% reported from São Paulo (southeast) [34]; 73.5% in Mato Grosso do Sul (midwest) [35]; 75.3% in Paraná (south) [36]; 77.2% in Bahia (northeast) [17]. However, data extracted from previous studies in Brazil should be analysed with some caution, once they were limited, isolated, and usually reflect the results from small towns and/or of restricted groups (day-care centres, schools, indigenous tribes, small hospitals, fishing villages, *etc*.). Attention should also be given to studies conducted in other countries: Argentina (78.3%) in children living in a poor area [37]; Peru (66.3%) in orphanages [38]; Honduras (43.5%) in school going children [39]; Pakistan (52.8%) in children residing in slum areas [5]; and India (68%) in school going children [40].

In the present work, the most common pathogenic species detected were Complex *E*. *histolytica/E*. *dispar* (13.5%) and *G*. *lamblia* (8.1%). These two parasites are frequently found in Brazil [17,18,35,41]. However, detection *of G*. *lamblia* cysts is particularly alarming since these are resistant to conventional routine disinfectants, and are frequently found in sewage effluent and surface water [10]. In addition, individuals infected with *G*. *lamblia* are largely asymptomatic, and can spread the infection, contributing to high epidemic rates. Similarly, concern should also be given to the presence of *B*. *hominis* (12.7%), since its pathogenicity is still controversial [42]. In Minas Gerais (Brazil), Cabrine-Santos and colleagues [43] observed that 8% of participants with diarrhoea had only *Blastocystis* spp. (monoparasitism); suggesting that the parasite may have a pathogenic character.

Although soil-transmitted helminths (*A*. *lumbricoides*, *T*. *trichiura*, hookworms and *S*. *stercoralis*) are the most frequent parasites found in many countries [32, 39], they were not the predominant enteric parasites in this study. Probably these parasites cannot complete their life cycles due the absence of an adequate soil environment or the presence of road/sidewalk paving or a high construction index [16]. The low prevalence of *S*. *mansoni* infections was also observed. The transmission of *S*. *mansoni* is dependent on the presence of a water and an intermediate host snail, which may be not available in the areas of this study. According to the Brazilian Ministry of Health [44], the positive rate of *S*. *mansoni* in Rio de Janeiro State is 1.56%, making it the State with the lowest number of confirmed cases.

We noticed a positive association between intestinal parasites and the male gender. Similar results are observed in Brazil [43] and Iran [45,46], with a slightly higher prevalence of intestinal parasites in males than females. This association could be due to hygienic behaviours, specific occupations or even sexual activities, particularly among homosexuals, that may result in faecal/oral contact that subsequently leads to transmission of these agents [47,48].

Chemotherapy is one of the intervention strategies that reduce the incidence of intestinal diseases. Regular deworming with the drugs albendazole and mebendazole is the current global control strategy to reduce the prevalence of helminths, and is implemented in Brazil [44]. However, the deworming programmes are not effective against protozoa infections. In this study we clearly observed that the frequency of protozoan infections (87%) was much higher than that of helminths (13%). It is worth mentioning that nitazoxanide is an antiparasitic drug with a broad-spectrum activity against a variety of intestinal parasites (including protozoa and helminths). However, this product is not included in the list of pharmaceutical care products of the Unified Health System (SUS) in Brazil.

A number of individuals (141; 24.8%) were infected by multiple enteroparasites: 3.5% (5 of 141) of participants were infected with helminths, 80.9% (114 of 141) were infected with protozoa and 15.6% (22 of 141) by both. Polyparasitism remains persistent in the country: 18.4% of such cases were reported in São Paulo [34], 49.2% in Mato Grosso do Sul [35], 26.7% in Paraná [36], 51.2% in Bahia [17]. These works all showed the high frequency of protozoa. Polyparasitism had been observed in many countries [5,49,50]; for example, in Kenya, 7% of the study population was infected with multiple parasites [32], and Mejia Torres *et al*. [39] observed that 14.6% of children in Honduras were infected with more than one parasite.

This study confirms that the population has a high frequency of intestinal parasites, principally protozoa. Although the majority of parasites (62%) were non-pathogenic (*B*. *hominis*, *E*. *coli*, *E*. *hartmani*, *E*. *nana* and *I*. *butschilii*), it is important to note that these species have the same transmission path as other pathogenic protozoa, such as Complex *E*. *histolytica/E*. *dispar* and *G*. *lamblia*, indicating exposure to faecal contamination. The frequency of these parasites added to the high frequency of polyparasitism can be used as indicators of transmission through the faecal/oral route, thereby pointing to in the transmission of intestinal parasites via the supply of water for human consumption, or the ingestion of contaminated food.

Several authors have demonstrated the vulnerability of drinking water supply systems due contamination, which can lead to problems, such as the deterioration of water quality, which lead to the proliferation of pathogens, and, therefore, increase the risk of waterborne diseases [51,52]. Water for the citizens of the metropolitan region of Rio de Janeiro is provided by two principal supply systems, called Guandu-Piraí and Imunana-Laranjal. Both of these undergo the conventional treatment process, including coagulation, flocculation, filtration (granulated active carbon), fluoridation and chlorination [53]. Two companies carry out the operation and management of the water systems, one of which is public (State Company of Water and Sewage—CEDAE) and the other is a concession (Niterói Water). The Niterói Water Company only operates on the distribution of treated water, which is supplied by CEDAE from the water collected in the Imunana-Laranjal system. Although both systems operate satisfactorily, in agreement with Brazilian standards of technical quality and health [54], water distribution generally has problems inherent in the characteristics of the use and occupation of urban land in the metropolitan region of Rio de Janeiro, particularly in the municipalities and neighbourhoods with higher levels of social and economic inequality. In these areas, lack of access to collection services and sewage treatment leads to the contamination of the water supply network through cross connections and low pressure zones, thereby leading to the entry of sewage and rainwater into the system. This situation is exacerbated in neighbourhoods and slums located in higher areas, where the pressure in the network is insufficient to maintain a constant water flow, and, according to the Brazilian Standard, drinkability [55,56].

Although we did not directly investigate this matter, we know that in developing countries, such as Brazil, access to clean water, sanitation facilities and health infrastructure does not follow the population growth. Research conducted in two low-income communities of Campos dos Goytacazes (north of Rio de Janeiro State/Brazil) confirmed by water analysis that the entire underground water of the study area was contaminated and a high faecal contamination was detected in well water. The authors concluded that possibly inadequate sanitation, with sewage discharged directly into the soil in some points, visible leakage, along with inadequate, and negligent routine maintenance in some septic tank systems could certainly have contributed to the dissemination of diseases caused by parasites [57].

The high prevalence of intestinal parasitic infections is also closely related to the low level of education, the low household incomes family and improper hygienic practices [4,5,57]. This study evaluated the socio and economic conditions of the Rio de Janeiro population using an index of material deprivation (MDI) composed of three indicators (sanitation, income and education).

The Rio de Janeiro metropolitan area is comprised of many census tracts (CTs), very close together and with very different MDIs, resulting in the highly heterogeneous character of the Rio de Janeiro territory. For example, while the INI hospital was classified in the first deprivation quintile (q1), a large part of the resident population in its surroundings live in slums or very poor neighbourhoods and was classified in the last deprivation quintiles (q4 or q5). Such proximity of participants to slums makes them more likely to be infected with intestinal parasites. Clearly, the geospatial distribution of the detected intestinal parasitic infections was not random or homogeneous, but was influenced by the MDI and the proximity to INI. Discrepancy of the MDIs among the closest CTs reveals the need for a horizontal decision-making process, not only in the poorest areas of the municipality, but throughout their surrounding areas.

Improvements in sanitation systems, deworming and the creation of poverty reduction programmes (Bolsa Família and Favela Bairro Program) in Brazil have helped greatly to reduce the prevalence of intestinal parasites over the years, but much obviously remains to be done. Safe drinking water is a defining aspect of a developed country, and even today it is still a significant challenge to public health worldwide. Additionally, the lack of access to health services near their home forces individuals to travel great distances to demand medical treatment, and, in many cases, the lack or deficiencies in public transport prevents these people from accessing the medical units.

Access to medical care, preventative chemotherapy and improvements in water supply and sanitation are matters of urgency, and also require a massive education campaign for low and middle-income families. Water of good microbial quality must be continuously supplied to the households (avoiding storage, which is another factor for contamination), and thus preventing its theft. Diseases are not distributed occasionally or randomly, the existence of risk factors determines their distribution, so that constant and continuous monitoring is required. Efforts directed to build a health surveillance system are urgent for Rio de Janeiro, and require strategies based on: sanitary conditions, water supply, population vulnerability, socio-demographic and environmental factors such as age, gender, education, household characteristics and income. Knowing the geographical distribution of intestinal parasites in Rio de Janeiro population is an important first step that will assist in the decision-making process necessary to design effective preventive and control programs; however, more epidemiological studies are imperative. The ability to readily identify and reach individuals at highest risk of infection is an important aspect of parasitic disease control programmes.

## Supporting information

S1 Checklist STROBE checklist. (DOC)

S1 Table Data. (XLSX)

S2 Table Number of positive and negative participants to intestinal parasites by regions. (DOCX)

S1 Fig Population density of Rio de Janeiro state. (TIF)
