# Supplementary material for: Geospatial distribution of intestinal parasitic infections in Rio de Janeiro (Brazil) and its association with social determinants
Source: PLoS Negl Trop Dis. 2017 Mar 8;11(3):e0005445. doi: 10.1371/journal.pntd.0005445 (PMC5358884; doi:10.1371/journal.pntd.0005445)
Supplement: S1 Checklist — (DOC) [file pntd.0005445.s001.doc]

**S1. STROBE checklist.**

|  | Item No | Recommendation |
| --- | --- | --- |
| **Title and abstract** | 1 | (*a*) Indicate the study’s design with a commonly used term in the title or the abstract – *Page 1 “Geospatial Distribution of Intestinal Parasitic Infections in Rio de Janeiro (Brazil) and Its Association with Social Determinants”* |
|  |  | (*b*) Provide in the abstract an informative and balanced summary of what was done and what was found – *Pages 2 and 3* |
| Introduction | | |
| Background/rationale | 2 | Explain the scientific background and rationale for the investigation being reported – *Pages 5-7* |
| Objectives | 3 | State specific objectives, including any prespecified hypotheses – *Page 7 “The aim of this study was to estimate the number of individuals infected with intestinal parasites who attended a referral hospital located in Rio de Janeiro (Brazil), and to provide a detailed analysis of the geographical distribution. The study also looked at the influence of demographic variables, socio-economic status and environmental factors on the intestinal parasitic infections. This knowledge will be essential for the development of effective prevention and control strategies to eliminate or reduce intestinal parasitic infection.”* |
| Methods | | |
| Study design | 4 | Present key elements of study design early in the paper – *Pages 7-12* |
| Setting | 5 | Describe the setting, locations, and relevant dates, including periods of recruitment, exposure, follow-up, and data collection – *Pages 7-12* |
| Participants | 6 | (*a*) Give the eligibility criteria, and the sources and methods of selection of participants – *Pages 10* |
| Variables | 7 | Clearly define all outcomes, exposures, predictors, potential confounders, and effect modifiers. Give diagnostic criteria, if applicable – *Page 10-11* |
| Data sources/ measurement | 8* | For each variable of interest, give sources of data and details of methods of assessment (measurement). Describe comparability of assessment methods if there is more than one group – *Page 10-12* |
| Bias | 9 | Describe any efforts to address potential sources of bias – *N/A* |
| Study size | 10 | Explain how the study size was arrived at – *Page 10* |
| Quantitative variables | 11 | Explain how quantitative variables were handled in the analyses. If applicable, describe which groupings were chosen and why – *Page 11-12* |
| Statistical methods | 12 | (*a*) Describe all statistical methods, including those used to control for confounding – *Page 12* |
|  |  | (*b*) Describe any methods used to examine subgroups and interactions – *Pages 11-12* |
|  |  | (*c*) Explain how missing data were addressed –Any relevant missing data were listed in the paper. These were minimal and were not included in any analyses. |
|  |  | (*d*) If applicable, describe analytical methods taking account of sampling strategy – *N/A* |
|  |  | (*e*) Describe any sensitivity analyses – *N/A* |
| Results | | |
| Participants | 13* | (a) Report numbers of individuals at each stage of study—eg numbers potentially eligible, examined for eligibility, confirmed eligible, included in the study, completing follow-up, and analysed – *Table 2* |
|  |  | (b) Give reasons for non-participation at each stage – *N/A* |
|  |  | (c) Consider use of a flow diagram |
| Descriptive data | 14* | (a) Give characteristics of study participants (eg demographic, clinical, social) and information on exposures and potential confounders – *Table 3* |
|  |  | (b) Indicate number of participants with missing data for each variable of interest – *Tables 3, 6 ,7 and 8; and S2 Table* |
| Outcome data | 15* | Report numbers of outcome events or summary measures – *Tables 4-9 and Figures 2-4; and S2 Table* |
| Main results | 16 | (*a*) Give unadjusted estimates and, if applicable, confounder-adjusted estimates and their precision (eg, 95% confidence interval). Make clear which confounders were adjusted for and why they were included – *Tables 5, 8, 9 and Figures 2,3* |
|  |  | (*b*) Report category boundaries when continuous variables were categorized – *N/A* |
|  |  | (*c*) If relevant, consider translating estimates of relative risk into absolute risk for a meaningful time period – *N/A* |
| Other analyses | 17 | Report other analyses done—eg analyses of subgroups and interactions, and sensitivity analyses – *N/A* |
| Discussion | | |
| Key results | 18 | Summarise key results with reference to study objectives – *Pages 25-26* |
| Limitations | 19 | Discuss limitations of the study, taking into account sources of potential bias or imprecision. Discuss both direction and magnitude of any potential bias – *Pages 24-27* |
| Interpretation | 20 | Give a cautious overall interpretation of results considering objectives, limitations, multiplicity of analyses, results from similar studies, and other relevant evidence – *Pages 25-33* |
| Generalisability | 21 | Discuss the generalisability (external validity) of the study results – *Pages 29 and 33* |
| Other information | | |
| Funding | 22 | Give the source of funding and the role of the funders for the present study and, if applicable, for the original study on which the present article is based- Included as additional information in the submission form |

*Give information separately for exposed and unexposed groups.

**Note:** An Explanation and Elaboration article discusses each checklist item and gives methodological background and published examples of transparent reporting. The STROBE checklist is best used in conjunction with this article (freely available on the Web sites of PLoS Medicine at http://www.plosmedicine.org/, Annals of Internal Medicine at http://www.annals.org/, and Epidemiology at http://www.epidem.com/). Information on the STROBE Initiative is available at www.strobe-statement.org.
