# Supplementary material for: Geospatial distribution of intestinal parasitic infections in Rio de Janeiro (Brazil) and its association with social determinants
Source: PLoS Negl Trop Dis. 2017 Mar 8;11(3):e0005445. doi: 10.1371/journal.pntd.0005445 (PMC5358884; doi:10.1371/journal.pntd.0005445)
Supplement: S2 Table — (DOCX) [file pntd.0005445.s003.docx]

**S2 Table**. Number of positive and negative participants to intestinal parasites by regions.

| **Regions** | **Positive participants (n=569)** | | **Negative participants (n=2676)** | | **Total (n=3245)** |
| --- | --- | --- | --- | --- | --- |
|  | **N** | **%** | **N** | **%** |  |
| **RIO DE JANEIRO STATE** |  |  |  |  |  |
| **Metropolitan region** | 532 | 16.39 | 2315 | 71.34 | 2847 |
| Belford Roxo | 25 | 0.77 | 98 | 3.02 | 123 |
| Cachoeira de Macacu | 3 | 0.09 | 3 | 0.09 | 6 |
| Duque de Caxias | 56 | 1.73 | 197 | 6.07 | 253 |
| Guapimirim | 0 | 0 | 11 | 0.34 | 11 |
| Itaboraí | 4 | 0.12 | 17 | 0.52 | 21 |
| Itaguaí | 2 | 0.06 | 11 | 0.34 | 13 |
| Japeri | 2 | 0.06 | 8 | 0.25 | 10 |
| Magé | 7 | 0.22 | 25 | 0.77 | 32 |
| Maricá | 1 | 0.03 | 24 | 0.74 | 25 |
| Mesquita | 2 | 0.06 | 20 | 0.62 | 22 |
| Nilópolis | 13 | 0.4 | 32 | 0.99 | 45 |
| Niterói | 3 | 0.09 | 38 | 1.17 | 41 |
| Nova Iguaçu | 41 | 1.26 | 203 | 6.26 | 244 |
| Paracambi | 0 | 0 | 8 | 0.25 | 8 |
| Queimados | 2 | 0.06 | 13 | 0.4 | 15 |
| Rio Bonito | 0 | 0 | 7 | 0.22 | 7 |
| Rio de Janeiro | 332 | 10.23 | 1416 | 43.64 | 1748 |
| São Gonçalo | 15 | 0.46 | 87 | 2.68 | 102 |
| São João de Meriti | 22 | 0.68 | 85 | 2.62 | 107 |
| Seropédica | 2 | 0.06 | 6 | 0.18 | 8 |
| Tanguá | 0 | 0 | 6 | 0.18 | 6 |
|  |  |  |  |  |  |
| **Others municipalities** | 21 | 0.65 | 60 | 1.85 | 81 |
| Angra dos Reis | 1 | 0.03 | 1 | 0.03 | 2 |
| Araruama | 3 | 0.09 | 7 | 0.22 | 10 |
| Barra do Piraí | 0 | 0 | 1 | 0.03 | 1 |
| Barra Mansa | 2 | 0.06 | 2 | 0.06 | 4 |
| Cabo Frio | 0 | 0 | 1 | 0.03 | 1 |
| Campo dos Goytacazes | 0 | 0 | 1 | 0.03 | 1 |
| Comendador Levy Gasparian | 1 | 0.03 | 0 | 0 | 1 |
| Duas Barras | 0 | 0 | 1 | 0.03 | 1 |
| Iguaba Grande | 0 | 0 | 2 | 0.06 | 2 |
| Macaé | 1 | 0.03 | 4 | 0.12 | 5 |
| Mangaratiba | 2 | 0.06 | 5 | 0.15 | 7 |
| Miguel Pereira | 0 | 0 | 1 | 0.03 | 1 |
| Nova Friburgo | 0 | 0 | 2 | 0.06 | 2 |
| Parati | 1 | 0.03 | 0 | 0 | 1 |
| Paty de Alferes | 0 | 0 | 1 | 0.03 | 1 |
| Petrópolis | 0 | 0 | 3 | 0.09 | 3 |
| Pinheiral | 0 | 0 | 2 | 0.06 | 2 |
| Quissama | 0 | 0 | 1 | 0.03 | 1 |
| Resende | 0 | 0 | 1 | 0.03 | 1 |
| Rio Claro | 0 | 0 | 1 | 0.03 | 1 |
| Rio das Ostras | 0 | 0 | 5 | 0.15 | 5 |
| Santa Maria Madalena | 0 | 0 | 1 | 0.03 | 1 |
| Santo Antônio de Pádua | 3 | 0.09 | 0 | 0 | 3 |
| São Fidélis | 0 | 0 | 1 | 0.03 | 1 |
| São Francisco de Ibapoana | 0 | 0 | 1 | 0.03 | 1 |
| São José do Vale do Rio Preto | 0 | 0 | 2 | 0.06 | 2 |
| São Pedro da Aldeia | 1 | 0.03 | 2 | 0.06 | 3 |
| Saquarema | 3 | 0.09 | 4 | 0.12 | 7 |
| Teresópolis | 0 | 0 | 1 | 0.03 | 1 |
| Trajano Morais | 0 | 0 | 2 | 0.06 | 2 |
| Três Rios | 1 | 0.03 | 0 | 0 | 1 |
| Valença | 1 | 0.03 | 0 | 0 | 1 |
| Vassouras | 0 | 0 | 1 | 0.03 | 1 |
| Volta Redonda | 1 | 0.03 | 3 | 0.09 | 4 |
|  |  |  |  |  |  |
| **OTHERS STATES OF BRAZIL** | 0 | 0 | 9 | 0.28 | 9 |
| **UNKNOWN** | 16 | 0.5 | 292 | 9 | 308 |
