# Supplementary figures and images for: Geospatial distribution of intestinal parasitic infections in Rio de Janeiro (Brazil) and its association with social determinants
Source: PLoS Negl Trop Dis. 2017 Mar 8;11(3):e0005445. doi: 10.1371/journal.pntd.0005445 (PMC5358884; doi:10.1371/journal.pntd.0005445)

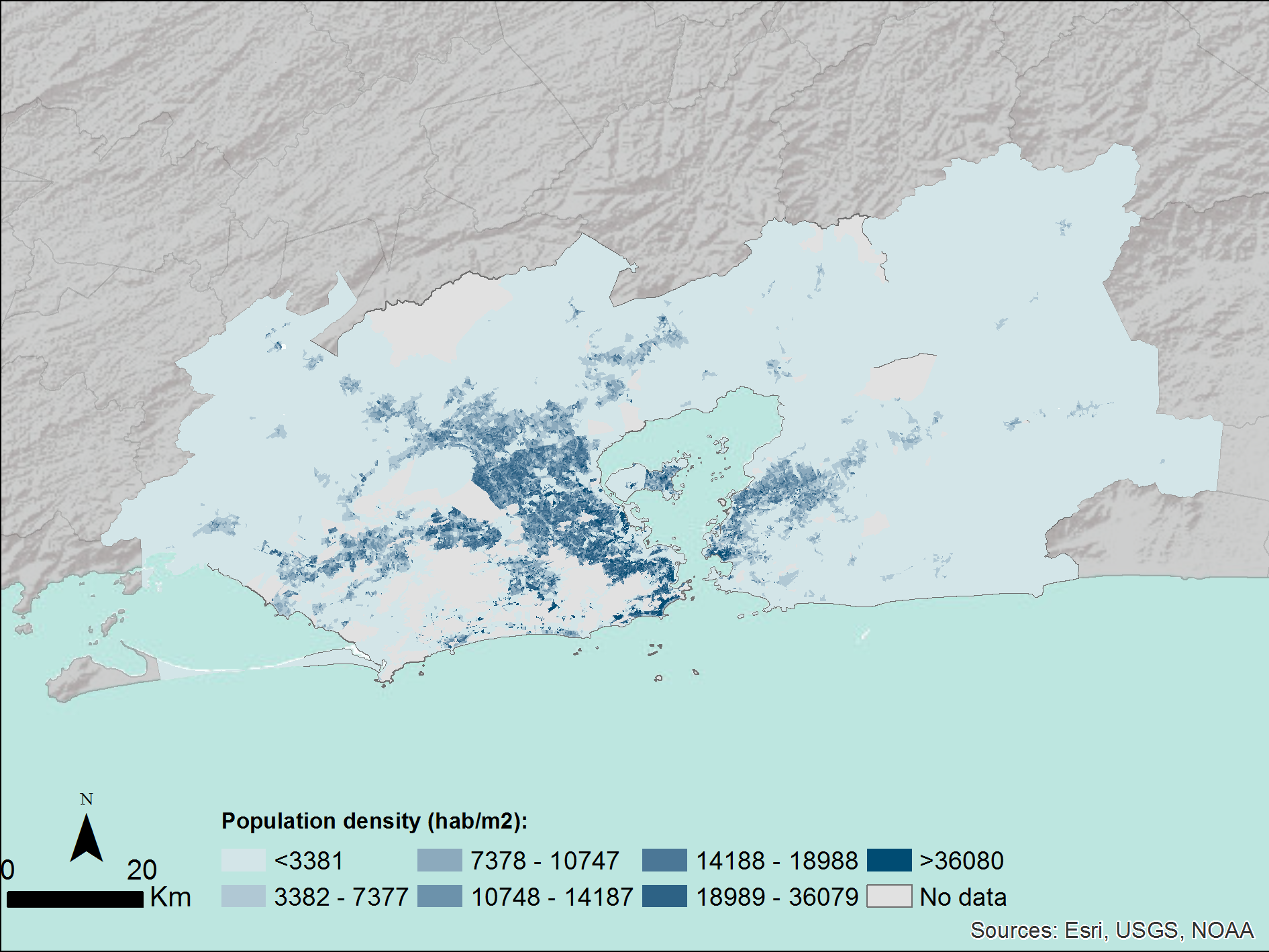

Supplement: S1 Fig — (TIF) [file pntd.0005445.s004.tif]
